# SiR–Hoechst is a far-red DNA stain for live-cell nanoscopy

**DOI:** 10.1038/ncomms9497

**Published:** 2015-10-01

**Authors:** Gražvydas Lukinavičius, Claudia Blaukopf, Elias Pershagen, Alberto Schena, Luc Reymond, Emmanuel Derivery, Marcos Gonzalez-Gaitan, Elisa D'Este, Stefan W. Hell, Daniel Wolfram Gerlich, Kai Johnsson

**Affiliations:** 1 Ecole Polytechnique Fédérale de Lausanne (EPFL), Institute of Chemical Sciences and Engineering (ISIC), Institute of Bioengineering, NCCR in Chemical Biology, 1015 Lausanne, Switzerland; 2 Institute of Molecular Biotechnology of the Austrian Academy of Sciences (IMBA), Vienna Biocenter Campus (VBC), Dr Bohr Gasse 3, 1030 Vienna, Austria; 3 Department of Chemistry—BMC, Uppsala University, 75123 Uppsala, Sweden; 4 Department of Biochemistry, NCCR in Chemical Biology, University of Geneva, 1211 Geneva, Switzerland; 5 Department NanoBiophotonics, Max-Planck-Institute for Biophysical Chemistry, Am Fassberg 11, 37077 Göttingen, Germany

## Abstract

Cell-permeable DNA stains are popular markers in live-cell imaging. Currently used DNA stains for live-cell imaging are either toxic, require illumination with blue light or are not compatible with super-resolution microscopy, thereby limiting their utility. Here we describe a far-red DNA stain, SiR–Hoechst, which displays minimal toxicity, is applicable in different cell types and tissues, and is compatible with super-resolution microscopy. The combination of these properties makes this probe a powerful tool for live-cell imaging.

## Abstract (web summary)

Existing DNA stains for live cell microscopy are either toxic, require illumination with blue light, or are not compatible with super-resolution microscopy. Here the authors develop SiRHoechst, a non-toxic far-red DNA stain that is compatible with super-resolution microscopy.

## Graphical abstract

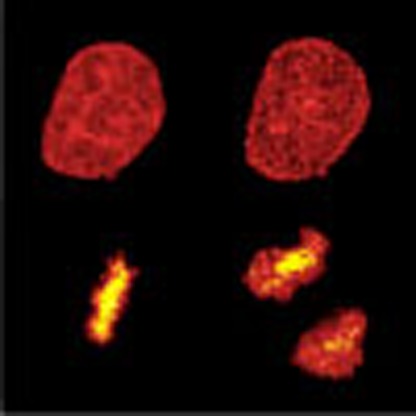

Many live-cell imaging applications rely on DNA stains as intracellular reference markers, to identify and track individual cells based on the nucleus or to visualize processes such as mitosis^1^. The ideal DNA stain for live-cell imaging would (i) show high selectivity, (ii) show minimal toxicity, (iii) be fluorogenic for wash-free imaging, (iv) be applicable in different cell types and tissues, (v) be excited by far-red light and (vi) be suitable for super-resolution microscopy. To the best of our knowledge, a stain that fulfils all these requirements has not been described yet. For example, the popular DNA minor groove-binder Hoechst 33342 is non-toxic when used at low concentrations but requires cell-damaging blue light illumination^2^ and is not compatible with super-resolution microscopy techniques such as stimulated emission depletion (STED) microscopy. In contrast, the anthraquinone-based intercalator DRAQ5 is a far-red DNA stain but is toxic at the concentrations used for live-cell microscopy^3^.

We previously demonstrated how the attachment of carboxylated silicon–rhodamine (SiR) derivatives to appropriate targeting ligands permits the generation of far-red probes for live-cell (super-resolution) microscopy of cellular proteins^4^,^5^. Furthermore, the bisbenzimide core of Hoechst 33342 has recently been exploited as a DNA-targeting ligand for green dyes^6^. Building on these findings we introduce here a bisbenzimide–SiR conjugate, SiR–Hoechst, and demonstrate its utility as DNA stain for live-cell (super-resolution) microscopy.

## Results

### Synthesis and characterization of SiR–Hoechst

**Figure 1:**
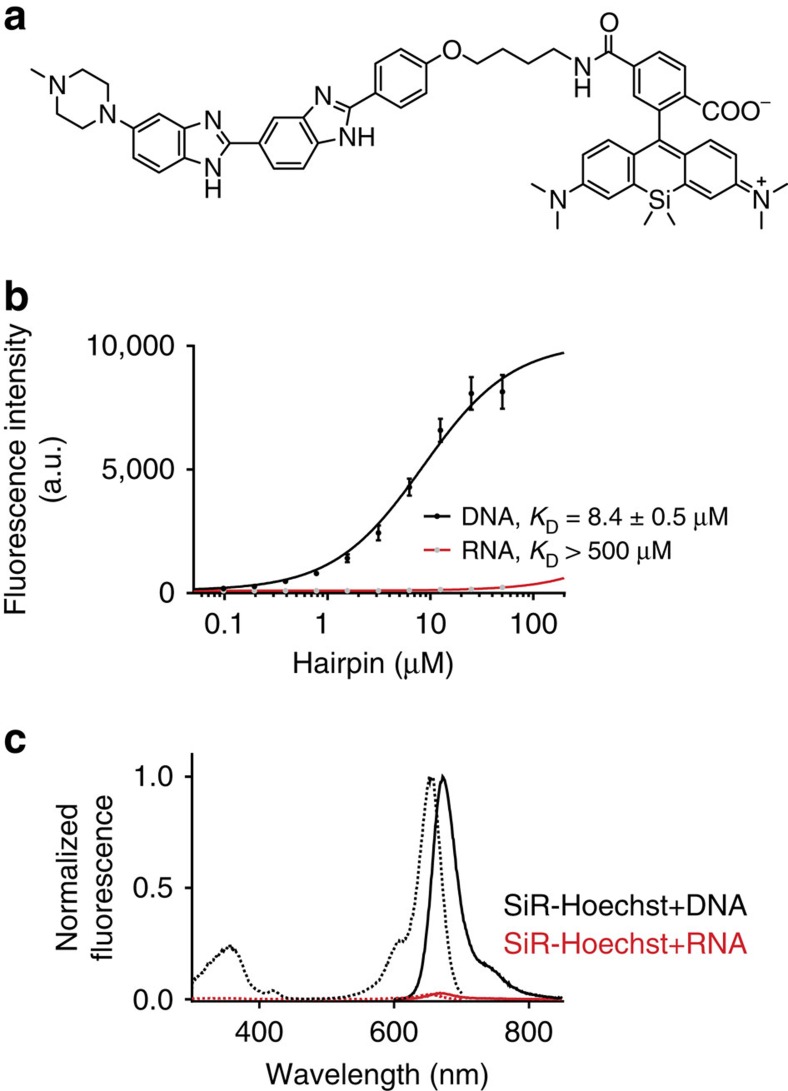
SiR–Hoechst. (**a**) Structure of the SiR–Hoechst. (**b**) Titration of 0.1 μM SiR–Hoechst probe with varying concentrations of hairpin DNA or hairpin RNA. Data points represent mean±s.d. of three independent replicates measured in triplicates. (**c**) Excitation spectra and absorbance spectra of 1 μM SiR–Hoechst in presence of 50 μM hairpin DNA or RNA. Determined excitation maximum at 652 nm and emission maximum at 672 nm.

Starting from commercially available building blocks SiR–Hoechst was synthesized in two steps with an overall yield of 25% (Fig. 1a and Supplementary Fig. 1). SiR–Hoechst binds to DNA with a *K*_D_ of 8.4 μM, which is about 1,000-fold lower than that of Hoechst 33342 for DNA but in line with that measured for other bisbenzimide derivatives^6^. SiR–Hoechst has no detectable affinity towards double-stranded RNA. As observed for other SiR-based probes, SiR–Hoechst is fluorogenic: Its fluorescence intensity at 670 nm increases about 50-fold on DNA binding while exciting at 640 nm (Fig. 1b,c). The increase in fluorescence of SiR–Hoechst on target binding is at least partially due to a shift of the equilibrium from the non-fluorescent spirolactone to the fluorescent zwitterion.

### Comparison of commercially available far-red DNA stains and SiR–Hoechst

**Figure 2:**
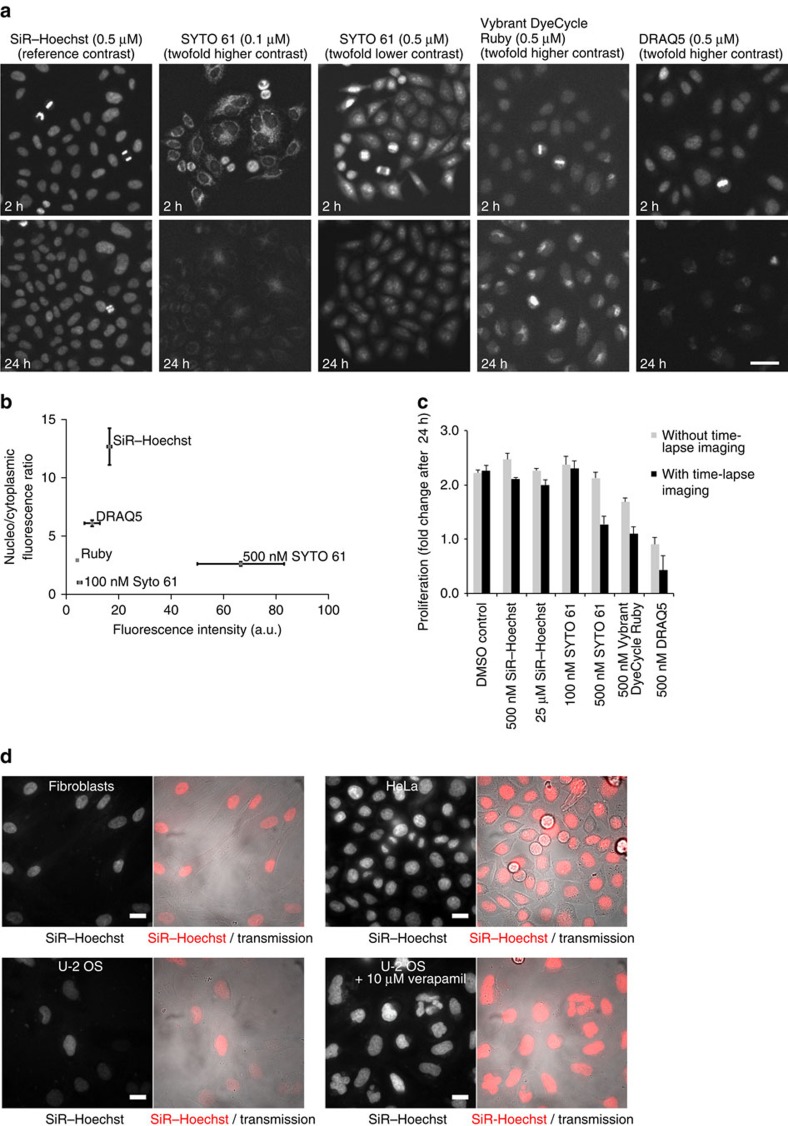
Live-cell imaging with SiR–Hoechst. (**a**) Long-term live-cell microscopy with SiR–Hoechst and other far-red DNA dyes. HeLa cells were imaged for 24 h on a wide-field epifluorescence microscope in presence of the dyes at the indicated concentrations, with a time lapse of 5.0 min. The contrast was linearly adjusted for the different dyes as indicated relative to the reference contrast used to display SiR–Hoechst (0.5 μM). Scale bar, 50 μm. (**b**) Quantification of nuclear-staining intensity and specificity. Nuclei were automatically segmented based on the H2B-mCherry channel and the mean fluorescence intensity was then quantified in the channel used for imaging far-red DNA dyes. The staining specificity was calculated as the ratio of nuclear mean fluorescence divided by cytoplasmic mean fluorescence quantified in a narrow area surrounding the nucleus. Spots indicate mean and bars indicate s.e.m. of three independent biological replicates; >1,108 cells per replicate. (**c**) Quantification of cell proliferation based on the last and the first frames of the live-cell movies shown in **a**. To assess phototoxicity caused by time-lapse imaging, separate wells in the imaging plate were exposed to fluorescence excitation light only at 0 and 24 h time points. Bars show mean±s.d. of three independent biological replicates; >76 cells per replicate. To quantify cells with no or low staining in the far-red channel, a stably expressed histone 2B (H2B)-mCherry was used as a reference chromatin marker. (**d**) Images of different cell types with 4 μM SiR–Hoechst. Scale bar, 20 μm.

To assess the applicability of SiR–Hoechst as a live-cell DNA dye, we cultured HeLa cells in medium containing 500 nM SiR–Hoechst. The cell population was homogenously and brightly stained about 30 min after addition of SiR–Hoechst and peak fluorescence levels were observed at about 90 min (Fig. 2a, Supplementary Movie 1).

We next compared the nuclear-staining specificity and potential cytotoxicity of SiR–Hoechst with three commercially available far-red DNA stains, SYTO 61, Vybrant DyeCycle Ruby and DRAQ5, using wash-free long-term live-cell epifluorescence microscopy of HeLa cells (Fig. 2a–c and Supplementary Movie 1). Of the tested far-red DNA dyes, SiR–Hoechst had highest specificity for nuclear staining above cytoplasmic background (Fig. 2a,b). At 500 nM, SiR–Hoechst yielded higher nuclear fluorescence intensity than DRAQ5 or Ruby, yet lower intensity than SYTO 61. SYTO 61, however, was limited by a very high cytoplasmic background signal, even when applied at much lower concentrations (Fig. 2a,b). Thus, SiR–Hoechst provides a bright and highly specific nuclear stain in live cells.

SiR–Hoechst did not impair cell proliferation within the 24-h measurement interval, up to the highest concentration tested (25 μM; Fig. 2c). DRAQ5, Ruby and SYTO 61, in contrast, were highly toxic at 500 nM (Fig. 2c). Without regular fluorescence excitation light exposure by time-lapse microscopy, the toxicity was reduced for DRAQ5 and Ruby, and it was undetectable for SYTO 61. This indicates that these three dyes substantially sensitize cells for phototoxicity, in contrast to SiR–Hoechst, which was imaged under identical conditions. In addition, DRAQ5 and Ruby strongly inhibited cell proliferation even in the absence of repetitive exposure to light by time-lapse microscopy. Thus, SiR–Hoechst outperforms the commercially available far-red DNA stains SYTO 61, Vybrant DyeCycle Ruby and DRAQ5 both in terms of labelling specificity as well as minimal toxicity.

### SiR–Hoechst stains various cell types

**Figure 3:**
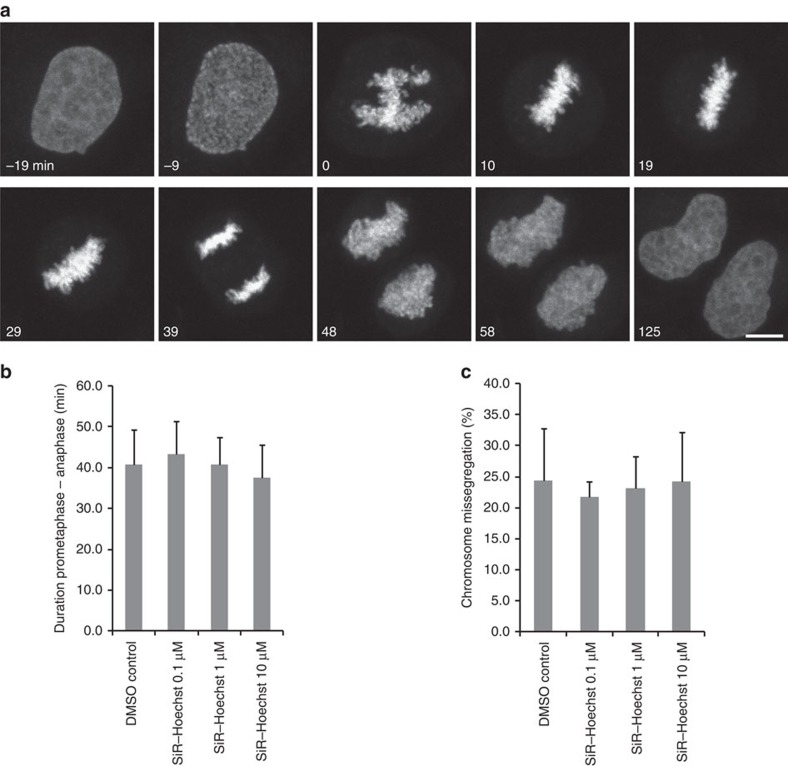
The 3D confocal time-lapse microscopy of cell division. (**a**) HeLa cells stained with 200 nM SiR–Hoechst were imaged over 3.4 h with a time lapse of 4.8 min. Images show maximum intensity projections of five *Z*-sections. To quantify control cells without staining in the far-red channel, a stably expressed histone 2B (H2B)-mCherry was used as a reference chromatin marker. Scale bar, 10 μm. (**b**) Mitotic duration was measured as the time from prometaphase onset until anaphase onset, based on visual inspection of the image data. (**c**) Chromosome missegregation was quantified as the number of anaphase events with lagging or bridged chromosomes divided by the total number of anaphase events, based on visual inspection. SiR–Hoechst was present in culture media throughout the entire imaging duration. Bars show mean±s.d. (*n*=3 independent biological replicates). DMSO, dimethylsulfoxide.

To further assess the applicability of SiR–Hoechst, we studied mitosis by confocal laser scanning three-dimensional (3D) time-lapse microscopy. SiR–Hoechst did not prolong mitotic progression and it did not increase the incidence of lagging or bridged anaphase chromosomes compared with unstained control cells that were assessed based on core histone H2B-mCherry as a reference chromatin marker (Fig. 3; Supplementary Movie 1). Given that mitosis is a highly sensitive process, we conclude that SiR–Hoechst is minimally toxic in live-cell microscopy applications even when applied for many hours.

We next investigated the applicability of SiR–Hoechst to other mammalian cell types. Populations of live human fibroblasts were homogeneously stained in the nucleus with very low cytoplasmic background (Fig. 2d). U-2 OS cells, however, were stained with variable intensities (Fig. 2d). A homogenous staining of U-2 OS cells by SiR–Hoechst could be achieved by co-incubation with the efflux pump inhibitor verapamil (Fig. 2d). This is in line with our previous observations that labelling efficiency with other SiR probes can be significantly improved through co-incubation with verapamil, which displays low toxicity in long-term live-cell imaging applications^4^,^7^,^8^(Mierzwa and Gerlich, unpublished observations).

To test whether SiR–Hoechst is suitable for whole organism imaging, we performed time-lapse confocal microscopy of *Drosophila* notum epithelium at the pupal stage. SiR–Hoechst enabled us to visualize chromosomes during cell divisions of epithelial cells for several hours without detectable signs of phototoxicity (Supplementary Fig. 2 and Supplementary Movie 2).

### Live-cell STED nanoscopy

**Figure 4:**
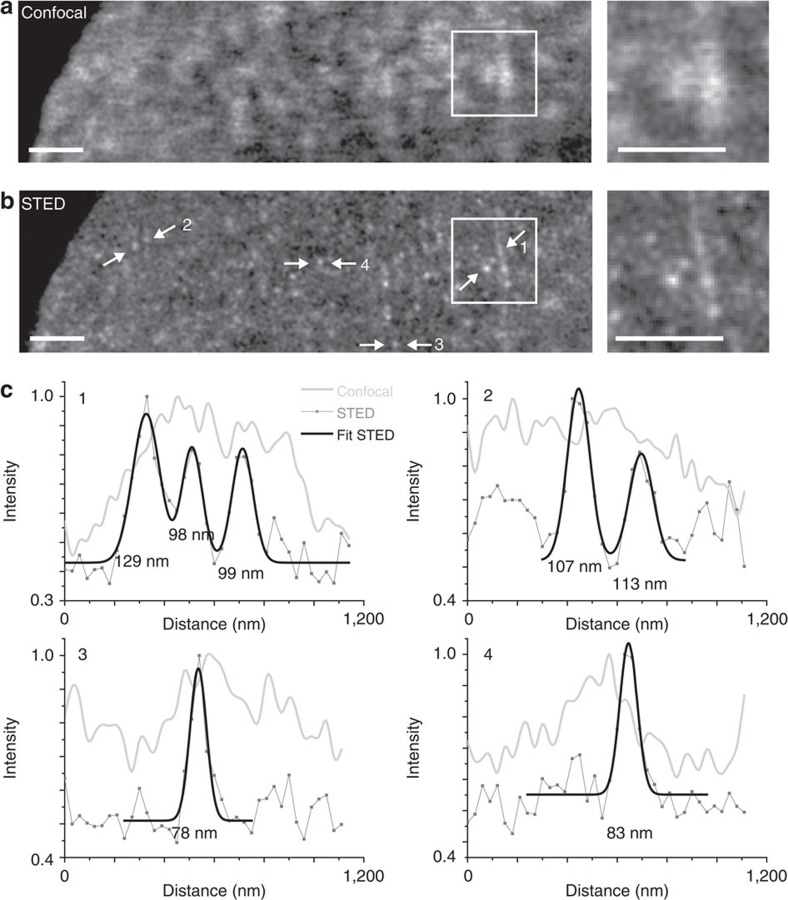
Comparison of confocal and STED imaging of HeLa cells stained with SiR-Hoechst. (**a**) Confocal and (**b**) STED images of living HeLa cell nuclei stained with 4 μM SiR-Hoechst for 2 h in phenol red-free medium. Both confocal and STED images were slightly smoothed. A part of the background in the STED image is caused by a weak but non-negligible two-photon excitation of Hoechst by the 775 nm STED beam. Scale bars, 1 μm. On the right, close-ups of the regions indicated with a box. (**c**) Examples of normalized fluorescence intensity profiles obtained in the regions indicated by the arrows in **b**. Profiles from the raw STED images were fitted to Gaussian distributions. Number corresponds to single measurement of full width at half maximum of the fitted peak.

Finally, we investigated whether SiR–Hoechst can be imaged with STED nanoscopy using a standard (commercially available) system equipped with a 775 nm STED beam. Staining of human primary fibroblasts or HeLa cells with SiR–Hoechst and subsequent live-cell STED nanoscopy revealed chromatin structures with a resolution well below 100 nm (Fig. 4). These proof-of-principle experiments underline the potential of SiR–Hoechst for DNA nanoscopy in intact cells. In contrast, Vybrant DyeCycle Ruby and DRAQ5 yielded high background signal caused by the 775 nm STED laser and are thus not compatible with standard STED systems (Supplementary Table 1 and Supplementary Fig. 3). SYTO 61 appears to be compatible, yet its higher toxicity and lower staining specificity (Fig. 2) makes it an inferior probe compared with SiR–Hoechst.

## Discussion

The coupling of the bisbenzimide core of Hoechst 33342 to carboxylated silicon–rhodamine yielded a new DNA stain, which is ideally suited for live-cell imaging. The far-red excitation and emission spectra of the probe minimize phototoxic effects and enable its use in combination with popular red- and green-emitting fluorescent protein tags^9^. SiR–Hoechst has higher nuclear-staining specificity in live cells than the popular far-red DNA stains SYTO 61, Vybrant DyeCycle Ruby and DRAQ5. Importantly, SiR–Hoechst was the only far-red DNA dye that we were able to image with high signal-to-noise ratio and undetectable toxicity on a confocal laser scanning microscope over extended periods of time. SiR–Hoechst is compatible with STED microscopy at the standard 775 nm wavelength, providing exciting opportunities to investigate how the 3D folding of DNA relates to the organization of core histones in intact nuclei^10^.

Our work furthermore underlines the utility of carboxylated SiR for the generation of far-red, fluorogenic probes for live-cell imaging. Prior work demonstrated that coupling SiR to ligands that specifically bind to proteins or insert in membranes can generate powerful fluorogenic probes for live-cell imaging^11^,^12^,^13^,^14^,^15^,^16^. Here we demonstrate that DNA-binding ligands such as the bisbenzimides can also be employed. Together, these data make a strong case for carboxylated SiR as first choice when coupling ligands to fluorophores for the generation of fluorescent probes for live-cell imaging.

In summary, the favourable spectroscopic properties, excellent biocompatibility and applicability to STED microscopy make SiR–Hoechst a powerful tool for the imaging of DNA in living cells and tissues.

## Methods

### Preparation of DNA or RNA hairpin

For the DNA- or RNA-binding studies hairpin-forming oligonucleotides 5′-CGCGAATTCGCGTTTTCGCGAATTCGCG-3′ (28 bp) or 5′-CGCGAAUUCGCGUUUUCGCGAAUUCGCG-3′ (28 bp), respectively, were purchased from Microsynth. Previously, this hairpin DNA has been used for structural studies of the interaction of Hoechst 33342 with DNA^17^. Thus, we purchased analogous hairpin RNA for binding studies. Synthetic oligonucleotides were dissolved in Tris-buffered saline (TBS; 50 mM Tris-HCl, 150 mM NaCl, pH 7.4) at 1 mM concentration. Hairpin formed by putting the tube with DNA or RNA solution into boiling water bath, which was slowly cooled down to room temperature.

### Determination of quantum yields

Quantum yields were determined on an Infinite M1000 spectrofluorometer (TECAN) in a 96-well plate. The absorption spectrum was recorded between 300 and 800 nm and the emission spectrum was recorded between 620 and 850 nm (excitation at 600 nm) for four different concentrations of probe alone or with 50 μM DNA in TBS (50 mM Tris-HCl, 150 mM NaCl, pH 7.4) with 1 mg ml^−1^ bovine serum albumin (BSA). Absorbance at 650 nm versus fluorescence at 670 nm was plotted. The ratio of the obtained slope values of probes to the free dye (SiR-carboxyl) slope value multiplied by the measured quantum yield of free dye gave the relative quantum yields reported in Supplementary Table 1.

### Determination of *K*_D_

*K*_D_ measurements were performed by titrating the SiR–Hoechst (100 nM in 150 mM TBS with 1 mg ml^−1^ BSA) with increasing concentrations of the 28-bp hairpin DNA or its RNA analogue in a 96-well plate and measuring the increase in fluorescence (*λ*ex=640 and *λ*em=670 nm) on a plate reader after 2–3 h incubation at room temperature. The *K*_D_ values were determined by plotting the emission intensity versus the DNA or RNA concentration and fitting the curve in Graphpad Prism 6 to the ‘one site—specific binding' function. Measurements performed three times, each time technical triplicates were measured.

### Cell preparation for microscopy

All the used cell lines were cultured in DMEM (Life Technologies, catalogue no. 61965-059) supplemented with 10% foetal calf serum (FCS) (FCS, PAA, catalogue no. A15-151) and pen/strep (100 units per ml and 100 μg ml^−1^, respectively, Biochrom AG, catalogue no. A2213) at 37 °C and 5% CO_2_. For STED imaging, cells were stained with the probes at 37 °C in HDMEM (phenol red-free DMEM—Invitrogen, catalogue no. 31053-028—buffered with 10 mM HEPES) supplemented with 10% FCS and pen/strep. Imaging was performed in HDMEM buffer with 10% FCS. For regular microscopy cells were stained in DMEM growth medium at 37 °C supplemented with 10% FCS.

A HeLa (‘Kyoto' strain) cell line stably co-expressing H2B-mRFP and MyrPalm-mEGFP^18^, was used for live-cell microscopy experiments. H2B-mRFP was imaged as a reference marker to quantify cell proliferation, mitotic duration and chromosome missegregation in the control cells (dimethylsulfoxide) that were not stained with SiR–Hoechst. HeLa cells were cultured in DMEM supplemented with 10% (v/v) fetal bovine serum (FBS), 1% (v/v) penicillin–streptomycin (Sigma), 0.5 μg ml^−1^ puromycin and 500 μg ml^−1^ G418. For live-cell imaging experiments, cells were grown either in LabTek II chambered coverslips (ThermoScientific) or 96-well plastic-bottom plates (μclear; Greiner Bio-One Ltd.), in DMEM containing 10% (v/v) FBS and 1% (v/v) penicillin–streptomycin, but without riboflavin and phenol red to reduce background fluorescence. For wide-field and confocal time-lapse imaging, SiR–Hoechst, SYTO 61 (Life Technologies) and Vybrant DyeCycle Ruby (Life Technologies) were added between 30 min and 2 h before imaging at the concentrations as indicated in the main text.

### Preparation of *Drosophila* cells

Nota of *w*^*1118*^; *UAS>mRFP-Pon*; *Neur>Gal4 Jupiter-GFP/TM6B* flies expressing Jupiter-GFP, a microtubule marker, were dissected 16 h after puparium formation at 25 °C in Clone 8 medium^19^. Dissected nota were incubated in Clone 8 medium enriched with 1.6 μM SiR–Hoechst for 30 min at room temperature, embedded in a fibrinogen clot to diminish tissue movements during fast 3D image acquisition^19^, and imaged in Clone 8 medium enriched with 1.6 μM SiR–Hoechst.

### Wide-field microscope set-up

Imaging of stained cells was performed on Leica DMI6000B microscope equipped with a Leica HCX PL APO × 100 1.47 oil objective and a Hamamatsu-C9100 EM-charge-coupled device camera (512 × 512 pixels) (Fig. 2d). The following dichroic mirror and filters were used for SiR signal detection: excitation BP 635/30, emission BP 700/72 and dichroic mirror at 650 nm. *Z*-stacks with voxel size of 240 × 240 × 692 nm were acquired and images were presented as maximal intensity projections.

Automated long-term time-lapse imaging with wide-field epifluorescence microscopy (Fig. 2a) was performed on a Molecular Devices ImageXpressMicro XL screening microscope, controlled by in-house-developed Metamorph macros^20^, using a 10 × 0.5 numerical aperture (NA) S Fluor dry objective (Nikon) and reflection-based laser autofocus. The following dichroic mirror and filters were used for SiR signal detection: excitation BP 640/30, emission BP 690/50 and dichroic mirror at 660 nm. A microscope stage incubator was used to maintain cells in humidified atmosphere of 5% CO_2_ at 37 °C.

### Confocal microscope set-up

For laser scanning confocal imaging (Figs 2a and 3a and Supplementary Movie 1), a customized Zeiss LSM780 microscope equipped with a × 40 1.4 NA oil DIC Plan-Apochromat objective (Zeiss) was used, controlled by ZEN 2011 software and an autofocus macro (AutofocusScreen) kindly provided by J. Ellenberg (European Molecular Biology Laboratory, Heidelberg). The following laser illumination and fluorescence emission detection was used for SiR imaging: 633 nm laser excitation, emission detected on 32-channel gallium arsenide phosphide (GaAsP) detector array at wavelengths between 647 and 691 nm. During imaging, a humidified atmosphere of 5% CO_2_ and 37 °C was provided by a European Molecular Biology Laboratory incubation chamber.

### Spinning disk confocal microscope set-up

Imaging of *Drosophila* notum epithelium (Supplementary Fig. 2 and Supplementary Movie 2) was performed using a 3i Marianas spinning disk confocal set-up based on a Zeiss Z1 stand, a × 63 PLAN APO NA 1.4 objective and a Yokogawa X1 spinning disk head followed by a × 1.2 magnification lens and an Evolve EMCCD camera (Photometrics). Jupiter-GFP was detected using a 50-mW 488-nm excitation laser and a 520/30 bandpass emission filter. SiR–Hoechst was detected using a 50-mW 633-nm excitation laser and a 647-nm long pass filter. Channels were acquired sequentially. Fast *Z*-stack acquisition of entire notum (66.5 μm by 0.5 μm increment) was obtained using a piezo stage (Mad City Labs, Madison, WI), and the resulting stack was projected using maximum intensity projection.

### STED microscope set-up

The images shown in Fig. 4 were taken on an Abberior Instruments QuadSCAN STED microscope, equipped with a 775 nm STED laser, a 640 nm excitation line and avalanche photodiode (APD) gated detection.

The images shown in Supplementary Fig. 3 were taken on Leica SP8 gSTED × 3 microscope equipped with HC PL APO CS2 × 100/1.40 oil objective and 775 nm STED laser. Excitation white light laser was set to 633 nm and signal was detected by HyD detector set to 650–700 nm interval with 0.3–6.0 ns time gating. Pinhole was set to 1 a.u., images were acquired with three times line averaging and pixel size in *xy* plane was 20 × 20 nm.

### Assessment of cytotoxicity and labelling specificity of DNA dyes

Proliferation index, mitotic duration and chromosome missegregation were scored by visual inspection of time-lapse movies. Proliferation was calculated as the ratio of all live cells in the last movie frame (24 h) divided by all live cells from the first movie frame. Mitotic duration was defined as the interval from prometaphase onset until anaphase onset for all cells entering mitosis within the first 12 h of the experiment, and determined based on the chromatin morphology. The incidence of chromosome missegregation was calculated by dividing the number of anaphase cells with lagging or bridged anaphase chromosomes by the total number of anaphase cells.

DNA labelling specificity was quantified 2 h after adding the different dyes by automated image analysis using the open-source CellCognition software^20^. Cell nuclei were automatically segmented in five consecutive image frames per experimental condition by local adaptive thresholding in the H2B-mCherry channel. Mitotic cells and dead cells were excluded from analysis based on automated classification by supervised machine learning. The mean fluorescence was then measured in the far-red channel. To calculate the nucleo/cytoplasmic fluorescence ratio, a cytoplasmic region was defined for each cell as a 5 pixel wide rim around the nuclear segmentation mask, spaced at a distance of 1 pixel. Extracellular background fluorescence was manually measured and subtracted from intracellular mean fluorescence measurements. The nucleo/cytoplasmic fluorescence ratio was first calculated for individual cells to derive the mean nucleo/cytoplasmic fluorescence ratio of the cell population. The mean and s.e.m. was then calculated for each dye condition based on three independent biological replicates. Fluorescence cross-talk from the H2B-mCherry channel was very low (<2% of the signal detected in 500 nM SiR–Hoechst-treated cells), as assessed by measuring fluorescence intensity in dimethylsulfoxide-treated control cells.

## Additional information

**How to cite this article:** Lukinavičius, G. *et al.* SiR–Hoechst is a far-red DNA stain for live-cell nanoscopy. *Nat. Commun.* 6:8497 doi: 10.1038/ncomms9497 (2015).

## Supplementary Material

Supplementary Information Supplementary Figures 1-3, Supplementary Table 1, Supplementary Methods and Supplementary References

Supplementary Movie 1 High resolution time-lapse confocal imaging of Hela cells. Cells were stained with 200 nM SiR-Hoechst before imaging.

Supplementary Movie 2 SiR-Hoechst imaging in the Drosophila Notum. Time-lapse spinning disk confocal imaging of Drosophila notum epithelium showing Jupiter-GFP, a microtubule marker, and SiR-Hoechst. Maximum intensity projection of 133 planes with an increment of 0.5 μm (66.5 μm total). This movie corresponds to Supplementary Figure 1. Scale bar 10 μm.
