# Supplementary material for: SiR–Hoechst is a far-red DNA stain for live-cell nanoscopy
Source: Nat Commun. 2015 Oct 1;6:8497. doi: 10.1038/ncomms9497 (PMC4600740; doi:10.1038/ncomms9497)
Supplement: Supplementary Information — Supplementary Figures 1-3, Supplementary Table 1, Supplementary Methods and Supplementary References [file ncomms9497-s1.pdf]

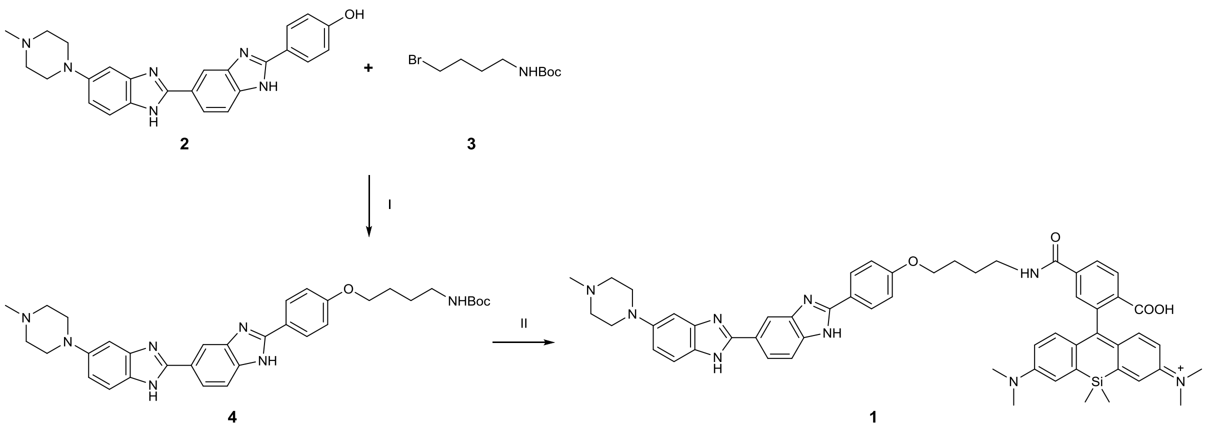

Supplementary Figure 1. Synthetic route of SiR-Hoechst. I) K_2CO_3, DMF, 60°C; II) a) TFA, DCM, rt b) SiR-NHS (Spirochrome), DIEA, DMSO, rt.

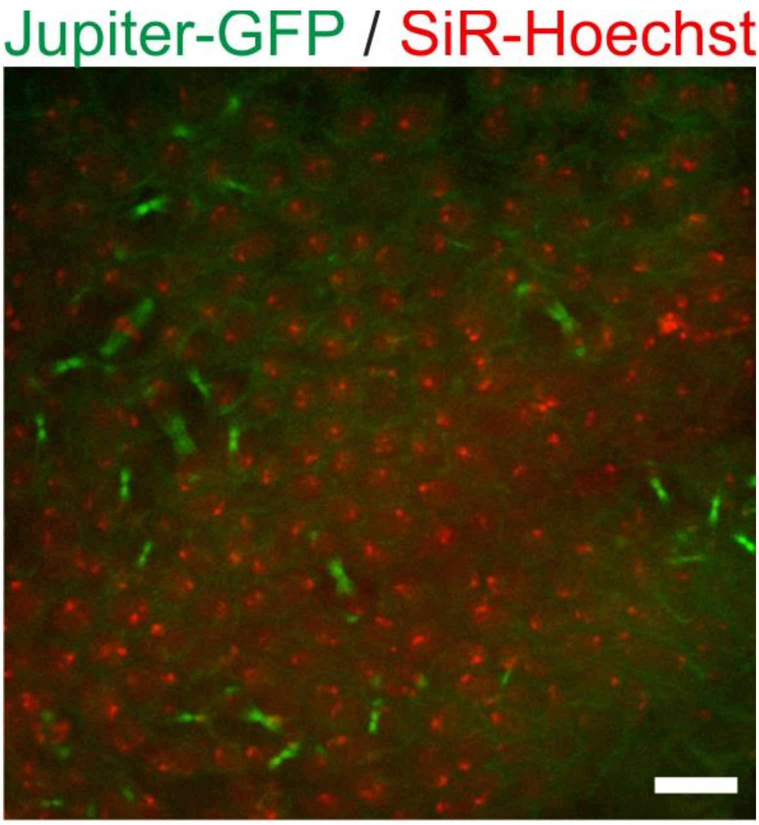

Supplementary Figure 2. SiR-Hoechst imaging in the Drosophila Notum. Spinning disk confocal imaging of Drosophila notum epithelium showing SiR-Hoechst and Jupiter-GFP, a microtubule marker. Image corresponds to maximum intensity projection of the whole tissue along the z-axis (66.5 µm). Scale bar: 10 µm.

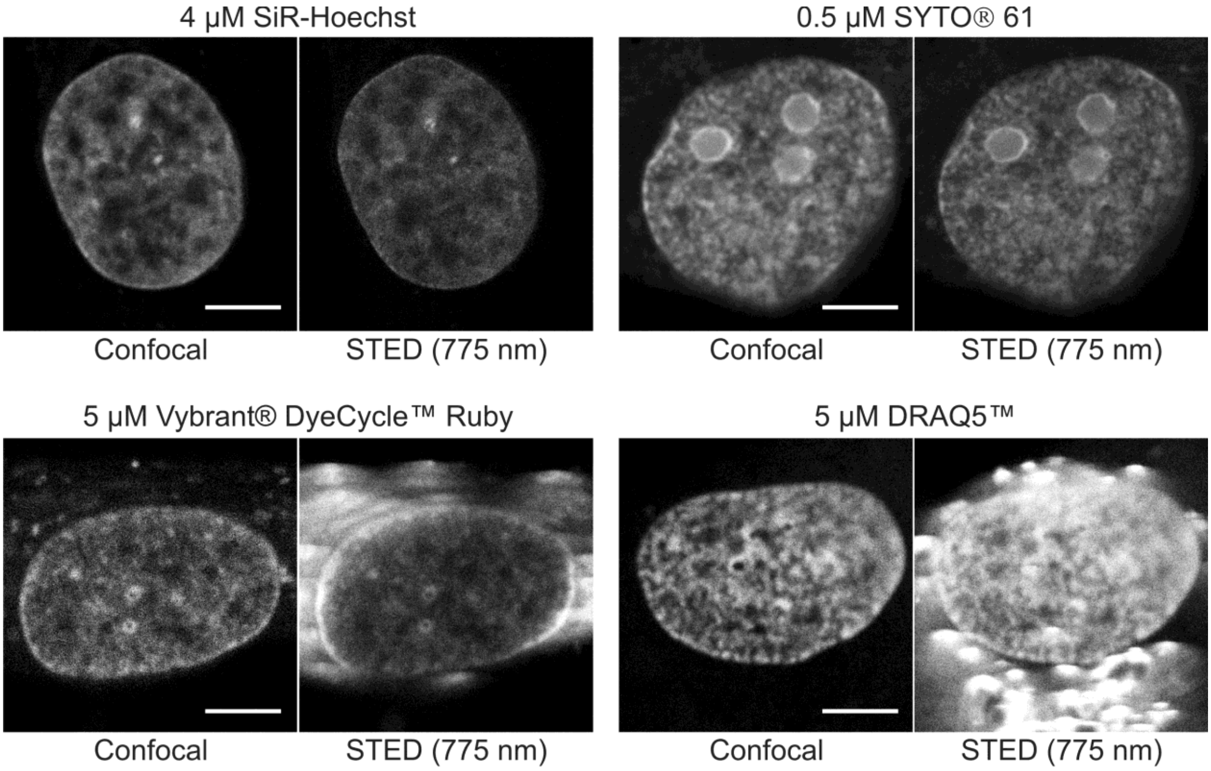

Supplementary Figure 3. Compatibility of far-red DNA stains with imaging on a commercial STED microscope equipped with a 775 nm STED laser. Living human primary fibroblasts nuclei stained with various cell permeable far-red DNA binding dyes for 1 – 2 h in growth medium. Both cofocal and STED images were aquired on Leica gSTED 3X microscope in the incubator set to +37°C. At least three cells were imaged per staining and shown images are representatives of typically obtained images. Scale bars 5 µm.

Supplementary Tables

Supplementary Table 1. Comparison of far red DNA stains and Hoechst 33342

Compound	Supplier	$\lambda_{Ex}/\lambda_{Em}$	ε	Φ	STED compatibility
SiR-Hoechst	N/A	652/672	100,000	0.17	+
Hoechst 33342	Sigma-Aldrich	350/461	46,000	0.38	N/A
SYTO® 61	Life technologies	628/645	43,000	0.18	+
Vybrant® DyeCycle™ Ruby	Life technologies	638/686	N/A	N/A	-
DRAQ5	Life technologies	647/681	21,000	N/A	-

Note: N/A – stands for “not available”. STED compatibility was tested using 633/640 nm excitation laser and 775 nm depletion laser. “+” – compatible with STED imaging; “-“ – not compatible with STED imaging.

Supplementary Methods

Origin of cell lines used in experiments

U-2 OS has been purchased form ATCC (ATCC® HTB-96™). Human primary dermal fibroblast has been puchased from Lonza (CC-2511). No transgene expressing HeLa ('Kyoto' strain) was kindly provided by prof. Pierre Gönczy. Live cell microscopy toxicity experiments performed with HeLa ('Kyoto' strain) cell line stably co-expressing H2B-mRFP and MyrPalm-mEGFP [1]. Drosophila notum imaging experiment performed using *w^{1118} ; UAS>mRFP-Pon ; Neur>Gal4 Jupiter-GFP / TM6B* flies expressing Jupiter-GFP [2].

General statistical analysis and experimental reproducibity

All measured parameters were measured at least in duplicates and presented as mean with standard deviation or standard error range as well as sample size indicated. In vitro replicates are measured on different days using the same setup. Each sample measured at least in technical duplicates. Cellular imaging experiments were performed at least twice on different days. Images at several different places of cell culture were acquired. If applicable, these parameters were indicated in the figure legends of the manuscript or supplementary information. Cell staining with probes was independently reproduced by the three groups of Daniel Gerlich, Stefan W. Hell and Kai Johnsson.

General considerations of chemical synthesis

All reactions were carried out in oven-dried glassware under nitrogen atmosphere, unless stated otherwise. Molecular sieves 4Å (MS4A) were purchased from Sigma Aldrich and activated by heating at 120°C for at least 24 hours. Chemicals were purchased from Sigma-Aldrich, Fisher Scientific, Merck, Alfa Aesar or Acros and used without further purification. Hoechst 33258 was purchased from Sigma as hydrochloride salt (bisBenzimide H 33258). SiR-NHS was purchased from Spirochrome (Stein am Rhein, Switzerland). Anhydrous solvents from Acros were used without further treatment or distillation. Flash column chromatography was performed with Merck silica gel (230-400 mesh). Reverse-phase preparative high-pressure liquid chromatography (Prep-HPLC) was performed on a Dionex system equipped with an UltiMate 3000 pump and an UV D170U UV-Vis detector for product visualization on a Waters SunFire Prep C18 OBD 5 µm 19×150 mm Column. Buffer

A: 0.1% v/v TFA in H_2O; Buffer B: acetonitrile; typical gradient was from 10% to 100% B within 22 minutes with 4 mL/min flow. LC-MS was performed on a Shimadzu MS2020 connected to a Nexera UHPLC system equipped with a Waters ACQUITY UPLC BEH C18 1.7 µm 2.1x50 mm column. Buffer A: 0.05%v/v HCOOH in H_2O Buffer B: 0.05%v/v HCOOH in acetonitrile. Analytical gradient was from 10% to 90% B within 5 min with 0.5 ml/min flow. Proton and carbon nuclear magnetic resonance (NMR) spectra were recorded at room temperature on a Bruker Avance-III 400 or on a Bruker DRX-600 equipped with a cryoprobe, with chemical shifts (δ) reported in ppm relative to the solvent residual signals. $CDCl_3$: δ_H 7.26 ppm, δ_C 77.16 ppm; DMSO-d6: δ_H 2.5 ppm, δ_C 39.5 ppm; CD_3OD: δ_H 3.31 ppm, δ_C 49.0 ppm. Coupling constants are reported in Hz. Multiplicity: s, singlet; br, broad; d, doublet, t, triplet; q, quartet; m, multiplet. High Resolution Mass Spectra (HRMS) were recorded on a Thermo Finnigan TSQ 7000. Solvent and reagent abbreviations: DMSO – dimethylsulfoxyde, DMF – dimethylformamide, DCM – dichloromethane, EtOAc – ethyl acetate, DIEA – diisopropylethylamine, MS4A – molecular sieves 4 Å, TFA – trifluoroacetic acid, DBU – 1,8-Diazabicyclo[5.4.0]undec-7-ene, Et_2O – diethylether, K_2CO_3 – potassium carbonate anhydrous, TSTU – O-(N-Succinimidyl)-N,N,N′,N′-tetramethyluronium tetrafluoroborate.

Synthesis of Hoechst-C_4-Boc 4

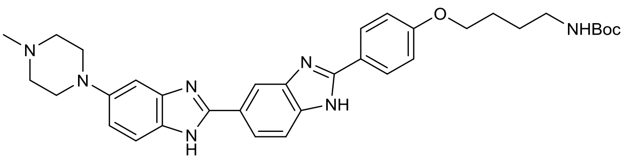

4

Hoechst-C_4-Boc **4** was obtained by alkylation of the commercial Hoechst 33258 dye. The free base of Hoechst 33258 was prepared by dissolving commercial Hoechst 33258 hydrochloride in H_2O (0.1 M, 1 eq) and adding a solution of 3.1 eq of 7 M K_2CO_3 (aq). The precipitate thus formed was isolated by centrifugation, washed with H2O and freeze dried. The resulting Hoechst 33258 base **2** (20 mg, 0.047 mmol, 1 eq) was suspended in dry DMF (235 µL, 0.2 M). K_2CO_3 (17.5 mg, 0.127 mmol, 2.7 eq) was added followed by 4-(Boc-amino)butyl bromide **3** (0.061 mmol, 1.3 eq). The reaction was heated at 60 °C for 18 h. The reaction mixture was then cooled to room temperature, diluted with MeCN:H_2O:AcOH 40:40:8 (880 µL) and purified by preparative HPLC (17.3 mg, 59% yield).

^{1}H NMR (400 MHz, MeOD) δ 8.29 (s, 1 H), 8.01 (d, 3 H, *J* = 8.7 Hz), 7.86 (d, 1 H, *J* = 8.6 Hz), 7.66 (d, 1 H, *J* = 9.0 Hz), 7.34 (dd, 1 H, *J* = 9.1, 2.2 Hz), 7.26 (d, 1 H, *J* = 2.0 Hz), 7.08 (d, 2 H, *J* = 8.9 Hz), 4.02 (t, 2 H, *J* = 6.1 Hz), 3.92 (m, 2 H), 3.67 (s, 2 H), 3.31 (m, 5 H), 3.22 (m, 2 H), 3.12 (t, 2 H, *J* = 6.9 Hz), 3.01 (s, 3 H), 1.80 (m, 2 H), 1.65 (m, 2 H), 1.45 (s, 9 H). ^{13}C NMR (101 MHz, MeOD) δ 164.3, 158.6, 155.1, 150.6, 150.0, 139.5, 137.3, 134.7, 130.6, 128.2, 124.5, 120.0, 119.3, 118.4, 116.6, 116.3, 115.6, 115.0, 100.9, 79.6, 69.2, 54.6, 43.6, 41.0, 28.8, 27.6, 27.5. HRMS (ESI) calcd for $C34H42N7O3^{+}$ $[M+H]^{+}$ 596.3344; found 596.3351.

Synthesis of SiR-Hoechst 1

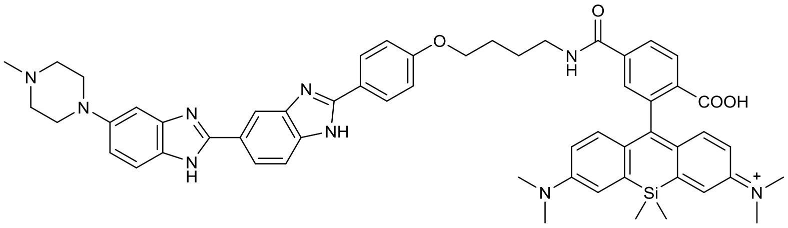

1

SiR-Hoechst **1** was synthesized using a 2 step reaction starting from the Boc-protected Hoechst derivative **4**. First, the Boc protecting group was removed by dissolving **4** in 20% TFA in CH_2Cl_2. The reaction mixture was stirred at rt for 5 min and then evaporated under a stream of N_2. The crude reaction mixture was dissolved in DMSO to a concentration of 10 mM (assuming full conversion) and used without further purification. SiR-NHS (Spirochrome) was dissolved in DMSO (200 uL, 10 mM, 1 eq) and treated with an equimolar amount of Hoechst-C_4-NH_2 in DMSO (200 uL, 10 mM, 1 eq), and DIPEA (3.5 µL, 10 eq). The reaction mixture was stirred at rt for 4 h. The reaction mixture was then diluted with 600 µL H_2O and 30 µL AcOH and purified by preparative HPLC (0.8 mg, 42% yield).

^{1}H NMR (400 MHz, MeOD) δ 8.39 (d, 1 H, *J* = 1.2 Hz), 8.26 (d, 1 H, *J* = 8.2 Hz), 8.12-8.08 (m, 3 H), 8.03 (dd, 1 H, *J* = 1.7, 8.5 Hz), 7.89 (d, 1 H, *J* = 8.5 Hz), 7.72-7.70 (m, 2 H), 7.38 (dd, 1 H, *J* = 2.2, 9.0 Hz), 7.31-7.29 (m, 3 H), 7.15 (d, 2 H, *J* = 8.9 Hz), 6.94 (d, 2 H, *J* = 9.5 Hz), 6.74 (dd, 2 H, *J* = 2.9, 9.5 Hz), 4.14 (t, 2 H, *J* = 5.8 Hz), 4.02-3.87 (m, 1 H), 3.76-3.58 (m, 2 H), 3.50-3.47 (m, 2 H), 3.42-3.16 (m, 4 H), 3.25 (s, 12 H) 3.02 (s, 3 H), 1.96-1.78 (m, 4 H), 0.64 (s, 3 H), 0.58 (s, 3 H). ^{13}C NMR (101 MHz, MeOD) δ 168.4, 168.2, 163.7, 163.1, 162.7, 162.4, 156.2, 154.5, 151.1, 150.6, 147.1, 141.4, 139.7, 139.4, 139.2, 135.0, 133.7, 131.3, 130.3, 130.1, 129.1, 128.9, 128.7, 123.8, 121.1, 120.5, 119.7, 119.4, 119.3, 116.5, 115.6, 115.6, 115.1,

101.2, 69.0, 54.7, 43.6, 40.9, 40.8, 30.8, 27.7, 27.1, -0.7, -1.6. HRMS (ESI) calcd for C56H60N9O4Si^{+} [M^{+}] 950.4538; found 950.4515.

LC-MS trace of SiR-Hoechst 1

HPLC trace at 654 nm and MS at 2.82 min

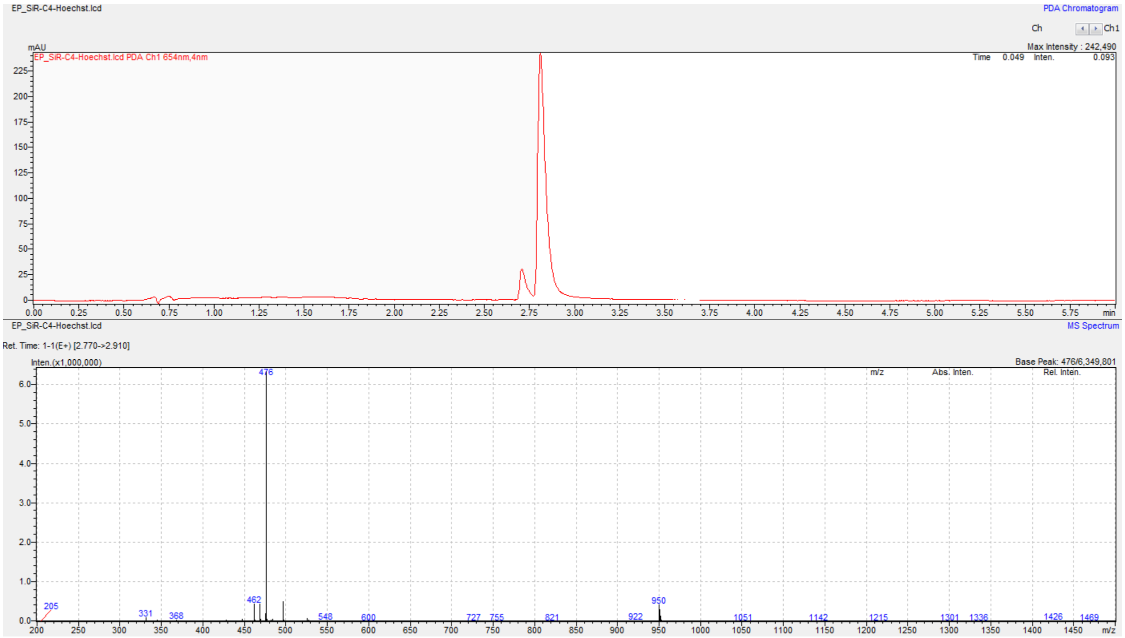

Supplementary References

1. Steigemann P*, et al.* Aurora B-mediated abscission checkpoint protects against tetraploidization. *Cell* **136**, 473-484 (2009).

2. Loubery S, Gonzalez-Gaitan M. Monitoring notch/delta endosomal trafficking and signaling in Drosophila. *Methods Enzymol* **534**, 301-321 (2014).
